# Single-walled carbon nanotubes as a reducing agent for the synthesis of a Prussian blue-based composite: a quartz crystal microbalance study†

**DOI:** 10.1039/d1na00739d

**Published:** 2021-11-24

**Authors:** Yosuke Ishii, Ayar Al-zubaidi, Yoshimitsu Taniguchi, Shinya Jindo, Shinji Kawasaki

**Affiliations:** Department of Life Science and Applied Chemistry, Nagoya Institute of Technology Gokiso-cho, Showa-ku Nagoya 466-8555 Japan a.al-zubaidi.052@nitech.jp ishii.yosuke@nitech.ac.jp kawasaki.shinji@nitech.ac.jp

## Abstract

We investigated the synthesis mechanism of Prussian blue (PB) crystals supported on single-walled carbon nanotubes (SWCNTs), by performing *in situ* quartz crystal microbalance (QCM) measurements to probe the change in the electrode mass during the reaction, and using photoirradiation at designated stages of the process. We found that in contrast to existing hypotheses, light irradiation played no role in the synthesis process of Prussian blue on SWCNTs. On the other hand, the number of electrons transferred per one mole of the obtained product, and the number of electrons transferrable from SWCNTs, calculated from the density of states (DOS) of the SWCNTs in the sample, both favor the hypothesis of the reaction being triggered by direct electron transfer from SWCNTs to Fe^3+^, which occurs because of the energy difference between the Fermi level of SWCNTs and redox potential of Fe^3+^ ions.

## Abstract 2

We investigated the synthesis mechanism of Prussian blue on single-walled carbon nanotubes, by performing *in situ* quartz crystal microbalance measurements and intermittent photoirradiation to probe the electrode mass change during the process.

## Graphical abstract

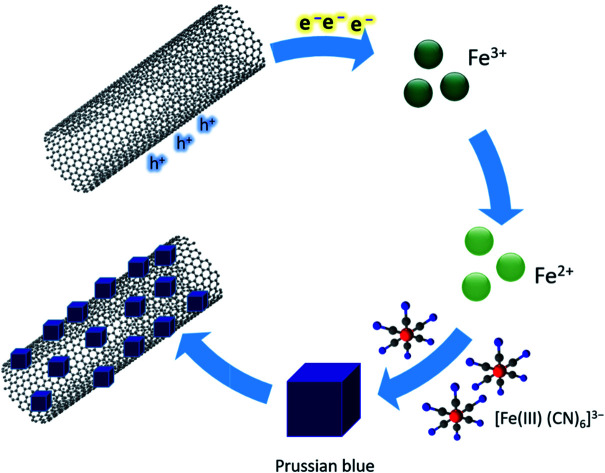

## Introduction

The market for lithium ion batteries continues to expand beyond portable electronics and electric/hybrid vehicles, requiring the device performance to sometimes accommodate a range of extreme temperatures spanning from −40 to 70 °C and wider.^1–3^ In particular, the use of lithium ion batteries in cold climates, space missions, military and other low temperature applications emphasizes the need to improve the performance of lithium ion batteries at sub-zero temperatures, at which the charging capability of the system drops considerably due to multiple possible factors, including most notably the deterioration in the lithium ion diffusion kinetics at low temperature.^4,5^ This problem sparked research efforts aiming to design electrode and electrolyte materials capable of delivering consistently high performance in wide temperature ranges, by modifying a number of existing battery materials or exploring new materials for the purpose.^6–9^

Among the materials being investigated towards that end are the class of cyano-based coordination materials called Prussian blue analogues (PBAs). Prussian blue is an iron centred mixed-valence coordination compound with the formula Fe_4_(iii)[Fe(ii)(CN)_6_]_3_·*n*H_2_O (*n* = 14–16), in which the Fe(iii) atom is coordinated to nitrogen and the Fe(ii) atom is coordinated to carbon in a basic face-centred cubic lattice structure.^10–12^ Prussian blue is an archetypical compound after which the family of PBAs is modelled with the general formula A_*x*_T[M(CN)_6_]·*n*H_2_O, in which A is an alkaline ion, T is a high-spin transition metal that coordinates with the N atoms, and M is a low-spin transition metal that coordinates with the C atoms.^13,14^ BPAs are attractive as both cathode and anode materials for alkaline ion-based batteries because their cage-like framework is suitable for reversible ion intercalation and maintains high structural integrity during cycling. They have been the focus of considerable research that demonstrated their promising energy and power performance at room temperature.^15–19^

The synthesis of PB is often a relatively easy and scalable one-pot process that mainly proceeds hydrothermally, by precipitation, or by electrodeposition.^13,20–23^ Among the three methods, precipitation is the most scalable and energy efficient, as it normally proceeds rapidly upon adding the metal ion salt to a solution of the cyanide ion salt precursor. The rapid reaction however hinders the product controllability and results in large random-morphology PB particles or aggregations with poor rate capability and cycling performance in energy applications. This issue can be overcome by adding coordination agents to reduce the reaction rate and allow the control of the product morphology, or by nucleating PB on the surface of a host material to ensure the formation of smaller well-distributed particles.^13^ The host material itself may contribute to improving the performance of the electrode. This is especially true for nanocarbon materials like graphene^24–27^ and carbon nanotubes^28–32^ that have been reported to impart high electric conductivity, nanoscale porosity, and a controllable surface structure and chemistry to the performance equation. Also importantly and in the context of low-temperature energy storage, the nucleation of PB on carbon nanotubes has been shown to result in a composite electrode that enjoys superior low-temperature power performance in a sodium ion battery, because carbon nanotubes provided a high active surface area for the electrode on the electrolyte side, and an excellent conductive path for electrons between the solid phase and the current collector.^28^

The role of nanocarbons has also been hypothesized by some studies to go beyond that of the conductive particle carrier that optimizes the PB particle size and electrode surface area. The synthesis reaction of PB on nanocarbons has been proposed to proceed by the carbon materials themself^33,34^ or the functional entities on their surface^35^ acting as a reducing agent by providing the electrons needed for the reaction to proceed. This hypothesis is difficult to confirm when graphene or multiwalled carbon nanotubes (MWCNTs) are used^30,36,37^ because the possibility and direction of electron transfer depend on the electronic structure of the material and the value of its Fermi level energy in relation to the redox potential of the synthesis reaction. This relation is difficult to map with graphene, a zero-band gap material that normally includes a number of chemical groups on its surface, or MWCNTs that are composed of a number of concentric tubes and overlapping electronic states. In addition, the crystallinity of the wall of any type of carbon nanotube is compromised, and its chemical composition is modified with functional groups that form on the surface when the sample goes through purification or functionalization,^29–33,37–39^ which adds another layer of complexity to the task of elucidating the synthesis mechanism.

In this study, we investigated the synthesis mechanism of PB on single-walled carbon nanotubes (SWCNTs) with high crystallinity and a narrow diameter range, and hence a relatively well defined electronic density of states (DOS). Experimentally, we probed the synthesis process using quartz crystal microbalance (QCM) measurements to track the change in the electrode mass resulting from the formation of PB particles on the surface of SWCNTs, and used light irradiation at designated stages of the synthesis process to test the hypothesis of spontaneous charge transfer *versus* the mechanism of photo-induced charge transfer proposed in some studies.^24,40,41^ The role of SWCNTs as an electron donor was also verified experimentally through the choice of the precursor material. The precipitation of PB is a solution reaction of either ferric chloride (FeCl_3_) with ferrocyanide ions [Fe(ii)(CN)_6_]^4−^, or ferrous chloride (FeCl_2_) with ferricyanide [Fe(iii)(CN)_6_]^3−^ ions.^13^ Should carbon nanotubes provide the electrons necessary for reducing Fe^3+^ and seeding Fe^2+^ ions on the surface of the tubes, the precursor combination of choice will be FeCl_3_ with [Fe(iii)(CN)_6_]^3−^.

To evaluate the performance of the composite at low temperature, the prepared composite was used as a cathode for a lithium ion battery charged and discharged at temperatures as low as −60 °C, and its energy storage behaviour was compared to crystalline PB and LiCoO_2_ that is commonly used as a lithium ion battery cathode.

## Experimental

The composite was synthesized using ferric chloride FeCl_3_ and potassium ferricyanide K_3_[Fe(CN)_6_] as PB precursors and single-walled carbon nanotubes (SWCNTs) produced using the eDips method,^42^ with an estimated mean diameter of 2.5 nm and carbon content of over 90% as supplied by the manufacturer. The electrolyte used in the electrochemical measurements was prepared by dissolving 1.0 M lithium bis(trifluoromethanesulfonyl)imide (LiTFSI) in the organic solvent 2-methyltetrahydrofuran (2-methyl THF). Table S1† gives the manufacturer information for all the materials used in the present study.

All the materials were used as received without further purification, except for the SWCNT sample that went through a purification treatment to remove any amorphous carbon and metallic catalyst particles on the surface of the tubes, an annealing treatment to improve the crystallinity of the tubes and cover any defects on their walls, and then a decapping treatment to open the end caps of the tubes.^43–45^

To synthesize the composite, first the SWCNT sample was dispersed in distilled water using a BRANSON SONIFIER 250 probe sonicator, and then 50 mL of a mixture containing 1.0 mM of FeCl_3_ and 1.0 mM of K_3_[Fe(CN)_6_] was added to the dispersion. The reaction mixture was left for 15 hours, and then the solid precipitate formed at the end of the reaction was collected and dried at 65 °C for 24 hours to obtain the composite that will be referred to as SWCNT@PB.

The structure of the composite was characterized along with a reference sample of crystalline PB. X-ray diffraction (XRD) measurements were performed using a Rigaku MINIFLEX diffractometer with a Cu Kα X-ray source (wavelength *λ* = 1.54 Å). The average tube diameter in the bare SWCNT sample and the SWCNT@PB sample was determined by simulating the XRD peaks of SWCNTs, and the obtained diameter values were used to determine candidate chiralities for the tubes in the sample, and obtain their DOS profiles using the Kataura plot.^46^

The composition of SWCNT@PB was determined from thermogravimetric analysis (TGA) using a SHIMADZU TGA-50 analyser operated at a heating rate of 5 °C min^−1^ under an air flow. Scanning electron microscopy (SEM) imaging and energy dispersive X-ray analysis (EDS) of the samples were performed using a JEOL JSM-7800F high resolution electric field emission scanning electron microscope. Transmission electron microscopy (TEM) imaging was performed using a JEOL JEM-z2500 transmission electron microscope. The sample to be probed was prepared by dispersing 1.0 mg in 5.0 mL ethanol using an AS ONE VS-70RS1 ultrasonic cleaner, and then depositing one drop of the dispersion on a STEM Cu150P copper microgrid and allowing it to dry at room temperature.

To prepare the SWCNT electrode for QCM measurements, the sample was first dispersed in *N*-methyl-2-pyrrolidone (NMP) using a BRANSON SONIFIER 250 ultrasonic probe, to obtain a solution 0.5 mg mL^−1^ in concentration. The solution was drop-cast onto a gold-coated quartz crystal and then left on a hot plate at 120 °C to dry. The QCM tracking of the SWCNT@PB synthesis took place in a dark room and as follows: first distilled water was drop-cast on the quartz substrate supporting the SWCNTs, and the frequency of the QCM was left to stabilize. The PB precursor solution was then added drop-wise, and the mass change was measured. After 10 minutes of adding the precursor solution, it was light-irradiated for 15 minutes to test the hypothesis of photo-induced synthesis. The solution was then monitored for 15 hours to allow the system sufficient time for stabilization. The change in the electrode mass was calculated from the change in resonance frequency using Sauerbrey's equation:1
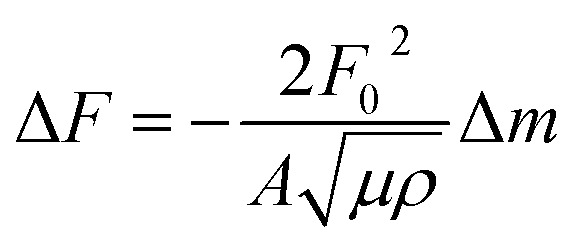
Here, Δ*F* is the frequency change of the crystal unit, *F*_0_ is the fundamental frequency, *A* is the area of the thin film electrode, *μ* (2.974 × 1011 g cm^−1^ s^−2^) is the elasticity of the crystal, *ρ* (2.648 g cm^−3^) is the density of the crystal, and Δ*m* is the mass change of the material attached to the electrodes.

The SWCNT@PB composite was used as a cathode material for a lithium ion battery to evaluate its low-temperature performance in comparison to that of crystalline PB and LiCoO_2_. The SWCNT@PB sample assumed the form of a free-standing paper, and was therefore used as an electrode without any binding or conductive additives. When crystalline PB or LiCoO_2_ was used, the electrode was prepared by mixing the active material (AC) with polyvinylidene fluoride (PVDF) as a binder and acetylene black (AB) as a conductive agent with an AC : AB : PVDF ratio of 4 : 4 : 2 for PB and 6 : 2 : 2 for LiCoO_2_, and then spreading the obtained paste onto an aluminum current collector sheet, and drying at 120 °C for a minimum of 1 hour under vacuum. The obtained electrode was used as the half-cell working electrode against lithium metal as the counter electrode, with 1.0 M LiTFSI/2-methyl THF organic solution as the electrolyte.

Constant current charge/discharge measurements were performed at room temperature and sub-zero temperatures using a TOSCAT-3200 (Toyo System) galvanostat. The voltage range was 2.3–3.3 V for PB and SWCNT@PB, and 3.0–4.0 V for LiCoO_2_. The room temperature measurements were performed in an argon-filled glove box, and the low temperature measurements were performed after assembling the half cell in an argon-filled atmosphere and then placing it inside a constant-temperature chamber (Espec SU-241) to perform the measurements. The device was charged and discharged at a current density of 25 mA g^−1^ relative to the mass of the active material in the working electrode (LiCoO_2_, crystalline PB, or PB in the SWCNT@PB composite). The charge/discharge capacity was calculated from the equation:2
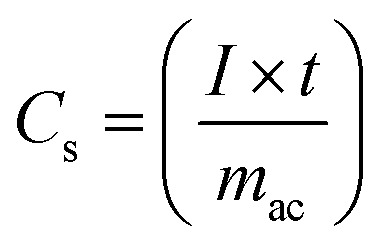
Here *C*_s_ is the specific capacity, *I* is the electric current, *t* is the time, and *m*_ac_ is the mass of the active material in the electrode. The rate capability was evaluated from charge/discharge measurements of the half cells at a current density of 25 mA g^−1^, 50 mA g^−1^, 100 mA g^−1^, 200 mA g^−1^, and then 25 mA g^−1^, with 5 cycles at each current density. The cycle stability was evaluated through charging and discharging the half-cells for 100 cycles at a current density of 100 mA g^−1^, and the coulombic efficiency was calculated from the ratio of the total charge obtained from the discharging process (*C*_d_) to that stored during the charging process (*C*_c_):3
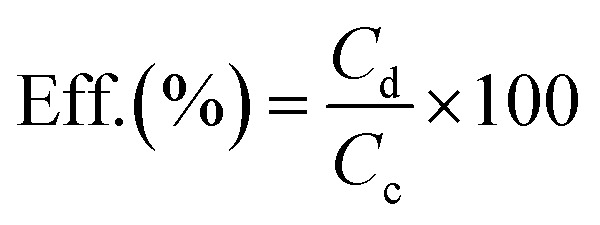


## Results and discussion

**Fig. 1:**
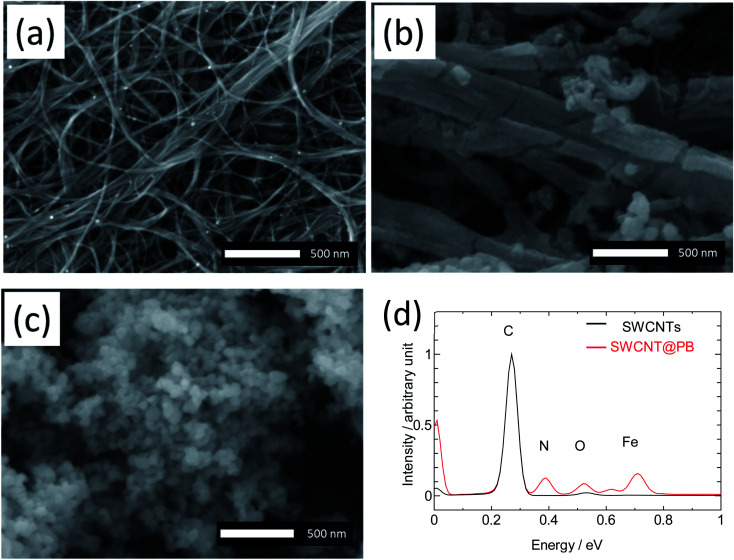
SEM images for pure SWCNTs (a), SWCNT@PB (b), and crystalline PB (c), and EDS analysis for SWCNTs and SWCNT@PB (d).

The nucleation of PB on the surface of SWCNTs results in a coating of small-size PB particles distributed on the surface of SWCNT bundles, which improves the charge storage efficiency of PB electrodes in energy devices. The morphology of the composite is seen in the SEM images in Fig. 1. Compared to the bare SWCNTs in Fig. 1a, the bundles of SWCNT@PB in Fig. 1b are covered with a layer of PB particles, as can be inferred from the bamboo segment-like deposits with smaller particle size compared to crystalline PB (Fig. 1c), and the bundle size that is larger than that of the bare SWCNTs. The EDS elemental analysis of SWCNT@PB (Fig. 1d) shows the characteristic peaks for Fe and N atoms in the sample.

**Fig. 2:**
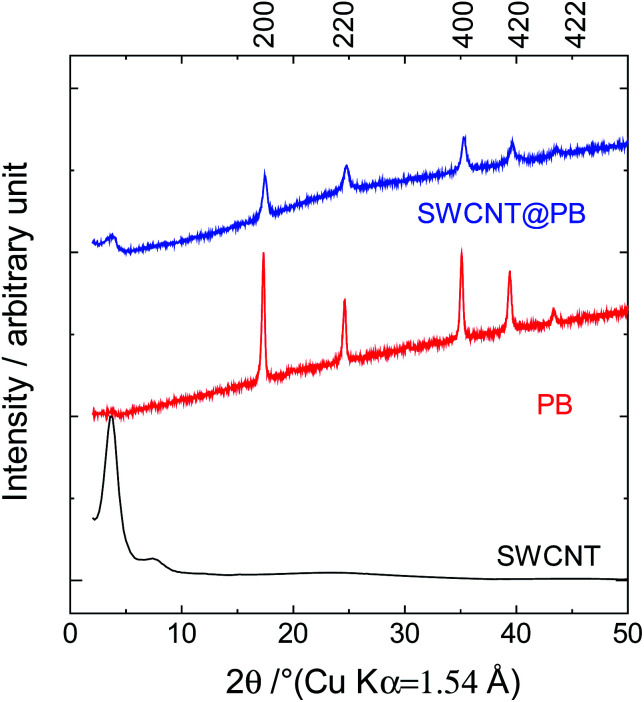
XRD patterns for crystalline PB, SWCNTs, and SWCNT@PB.

The characterization of the samples using X-ray diffraction (XRD) measurements resulted in the diffraction patterns shown in Fig. 2. The diffraction patterns for SWCNT@PB had similar peak positions to those of the crystalline PB sample, confirming the similarity in the structure. The 200 distance (the Fe–Fe distance) was calculated using Bragg's equation:

4 2*d*_200_ sin *θ* = *nλ*

The 200 interplanar distance for the crystalline PB was 5.12 Å, *versus* 5.09 Å for SWCNT@PB.

**Fig. 3:**
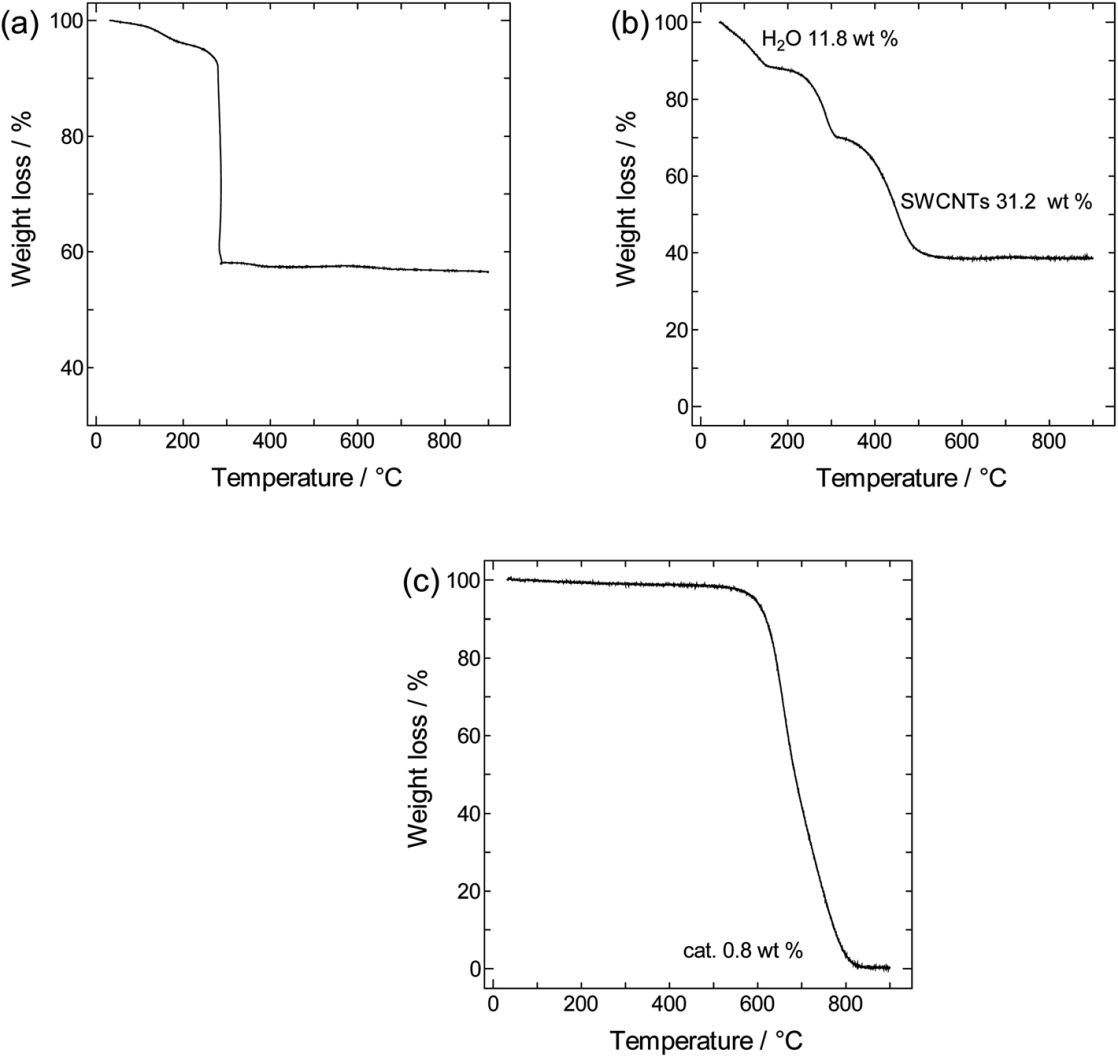
TGA curve for (a) crystalline PB, (b) SWCNT@PB and (c) annealed SWCNTs.

TGA curves obtained for crystalline PB, SWCNTs, and SWCNT@PB are shown in Fig. 3. Both crystalline PB (Fig. 3a) and SWCNT@PB (Fig. 3b) go through mass loss around 100 °C that is associated with the loss of zeolitic water, and around 260 °C, which is likely the combination of the loss of coordinated water and the decomposition of BP.^47,48^ Additional combustion stages are seen at 450 °C for SWCNT@PB, *versus* 580 °C for the annealed SWCNTs (Fig. 3c), both due to the decomposition of SWCNTs. After subtracting the SWCNT mass content (31.2%) and residual catalyst content (0.8%) and then normalizing the percentages to mass of the dry sample (calculated after the removal of H_2_O of 11.8%), a PB content of 63.7% in the composite was obtained, equivalent to a PB : SWCNT mass ratio of 1.8 : 1.

**Fig. 4:**
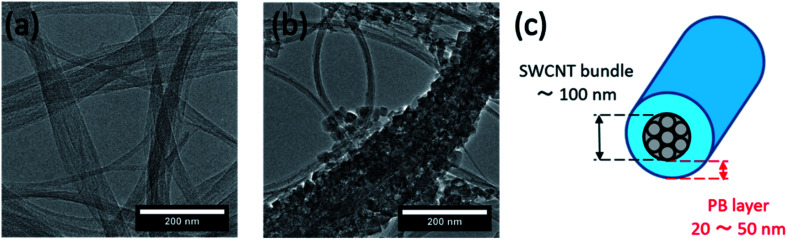
TEM images of (a) bare SWCNTs and (b) SWCNT@PB composite, and (c) core–shell model of the SWCNT@PB composite.

TEM images show a bundle of bare SWCNTs 50 nm to 100 nm in size in Fig. 4a, *versus* a core–shell-structured composite in Fig. 4b, in which a PB layer 20–50 nm in thickness is deposited on the surface of a SWCNT bundle around 100 nm in size. The TEM images did not show epitaxy in the growth of PB on the SWCNT bundles. We hypothesize that the difficulty in achieving epitaxial growth is caused by the fact that PB particles coat the bundles of SWCNTs, not the individual tubes, which should reduce the possibility of epitaxy due to intra-bundle heterogeneity in the diameters and chiralities of individual SWCNTs. In addition, the bamboo-like segments observed in the SEM images suggest simultaneous multi-site nucleation and poly-crystal formation rather the seamless single crystal expected in epitaxial growth. Nonetheless, the possibility of a seamless growth of a single crystal-like PB on SWCNTs remains an open question and an interesting one, especially in light of recent studies that reported the successful epitaxial growth of a number of crystalline materials on the surface of SWCNTs.^49,50^

For an independent estimation of the PB : SWCNT content ratio in the composite, we used the core–shell structural model (Fig. 4c) and size information obtained from SEM and TEM images. For a PB layer thickness of 30 nm, SWCNT bundle diameter of 100 nm, and tube length of 1 nm, the volume is 12 243 nm^3^ for PB *versus* 7859 nm^3^ for SWCNTs, resulting in a PB : SWCNT volume ratio of 1.56 : 1. Assuming a density of 1.80 g cm^−3^ for PB^12^ and 1.40 g cm^−3^ for SWCNTs,^51^ we obtained a PB : SWCNT mass ratio of 2 : 1, which is consistent with the ratio of 1.8 : 1 determined from TGA measurements.

To elucidate the synthesis mechanism and the potential role of SWCNTs in it, we performed QCM measurements to monitor the increase in the electrode mass due to the formation of PB particles, and identify the factors involved in the process. The QCM method probes the mass change through the changes it causes to the resonance frequency of the quartz crystal supporting the electrode.^52,53^

**Fig. 5:**
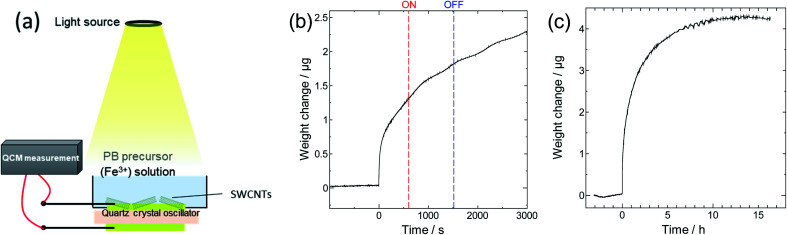
(a) QCM experiment (b) *in situ* tracking of electrode mass during synthesis and (c) extended time tracking of the synthesis process.

The *in situ* QCM setup and obtained results are shown in Fig. 5, where on and off in Fig. 5b denote the beginning and end of light irradiation, respectively, and the reference point (*t* = 0 seconds) is set as the time at which we started the addition of the PB precursor. Fig. 5b shows that the mass of the quartz crystal supporting SWCNTs increased sharply and immediately after the start of the addition of the PB precursor solution. The graph also shows that no additional change in mass occurred upon light irradiation, leading us to conclude that photo-induced charge separation does not seem to be involved in the Fe^3+^ reduction step of the composite synthesis. The duration for probing mass change was extended to 15 hours as seen in Fig. 5c, which shows that the system was allowed sufficient time for the precursors to react and for the material composition to stabilize. This again dismisses the possibility of later factors contributing to the product formation mechanism.

In a study by Choi *et al.*,^54^ the authors proposed the reduction of metallic Au and Pt ions to be triggered by spontaneous charge transfer from SWCNTs to the metallic ions, due to the difference between Fermi level energy for SWCNTs (0.5 V higher than the standard hydrogen electrode SHE) and the redox potential for those metals. In the case of PB, the synthesis reaction is:

5a A^+^ + Fe(CN)_6_^3−^ + Fe^2+^ → A_*x*_Fe(iii)[Fe(ii)(CN)_6_]_*y*_

Here A is an alkaline metal ion (in this case K^+^). For the formation of a pure molecule of insoluble PB, the equation is:

5b Fe(iii)(CN)_6_^3−^ + Fe^2+^ → Fe(iii)_4_[Fe(ii)(CN)_6_]_3_

When FeCl_3_ is used as the starting material, the reaction in eqn (5b) and the formation of PB will be dependent on and proceeded by the essential step of the reduction of Fe^3+^ to Fe^2+^:

6 Fe^3+^ + e^−^ ⇋ Fe^2+^; *E* = +0.771 V *vs.* SHE

The redox potential of Fe^3+^ (+0.771 V *vs.* SHE or −5.21 V *vs.* vacuum) is lower than the Fermi level of SWCNTs (−4.45 V *vs.* vacuum),^55^ which means that the hypothesis of spontaneous electron transfer from SWCNTs to Fe^3+^ should hold true for the formation of PB in the composite, provided of course that SWCNTs can offer the required supply of electrons for the reaction. This can be judged by estimating the number of electrons needed for the synthesis reaction *versus* the number of electrons available for transfer from SWCNTs. The former can be determined using the PB content determined from TGA measurements, and the latter may be estimated from the electronic density of states (DOS) of SWCNTs, which will depend strongly on the SWCNT chirality^56^ and in consequence its diameter. The exact chirality and DOS profile are hard to identify because a sample of SWCNTs will include a mixture of tubes of different chiralities. However, the narrow diameter range in our sample limits the number of those chiralities and allows the approximation of the DOS profile using one proxy chirality.

We calculated the number of electrons transferred in the synthesis reaction from the PB : SWCNT mass ratio obtained from TGA measurements (1.8 : 1), and the number of electrons required to synthesize 1 mol of PB. An ideal defect-free PB has the formula Fe_4_(iii)[Fe(ii)(CN)_6_]_3_, with a molecular mass of 859.23 g mol^−1^. The PB formula contains three Fe(ii) atoms, which means that according to eqn (5b) and (6), the formation of 1 mol of PB will necessitate the preliminary step of the reduction of three Fe^3+^ ions and hence the transfer of 3 electrons to the precursor solution.

**Fig. 6:**
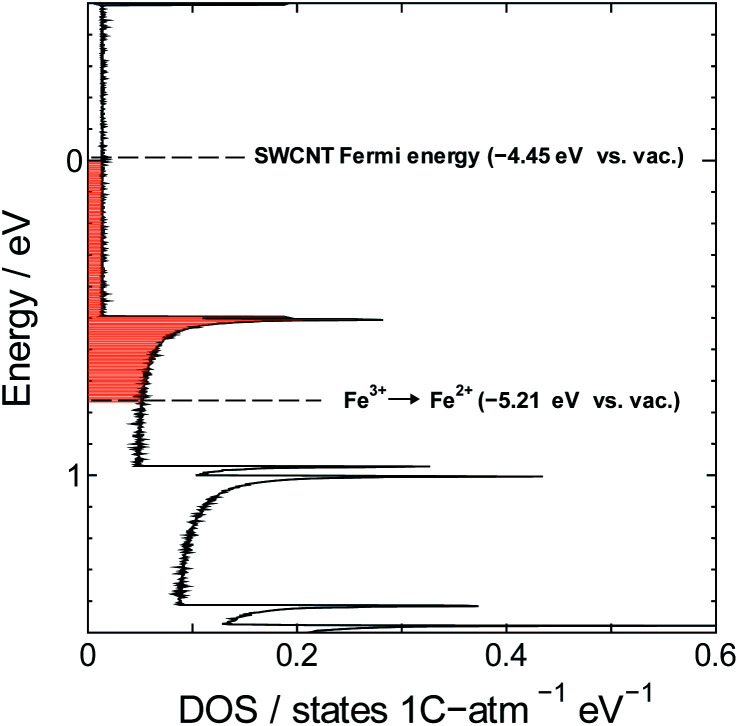
Position of the Fe^3+^ reduction potential relative to the DOS of (21,15) metallic SWCNTs.

Using the mass ratio obtained from TGA measurements, the total number of electrons transferred will be:
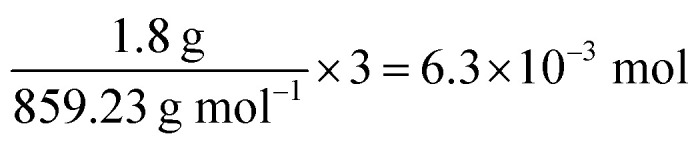


In a composite with a PB : SWCNT mass ratio of 1.8 : 1, the number of moles of carbon is:
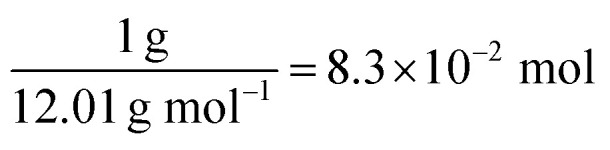


Therefore, the number of electrons needed per carbon atom is:
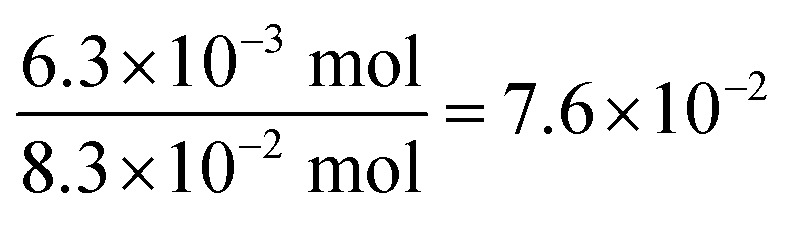


Next we estimate the number of transferrable electrons from SWCNTs. To do that we first used the Kataura plot to find candidate chiralities for SWCNTs with diameter size close to the mean diameter of our sample, and identified (21,16) semiconducting SWCNTs and (21,15) metallic SWCNTs as proxy chiralities for the calculations (DOS profiles shown in Fig. S1†). The number of transferrable electrons in SWCNTs will be that in the energy range between the Fermi level of SWCNTs (−4.45 V *vs.* vacuum)^55^ and the redox potential of Fe^3+^ (+0.771 V *vs.* SHE or −5.21 V *vs.* vacuum). The resulting difference of 0.76 V should promote electron transfer from SWCNTs to Fe^3+^ as shown in Fig. 6.

We used the trapezoidal formula to integrate the density of states over the indicated energy interval, and obtained a value of 5.0 × 10^−2^ electron per carbon atom for the (21,16) semiconducting SWCNTs and 5.3 × 10^−2^ electron per carbon atom for the (21,15) metallic SWCNTs. The two values are close to the value of 7.6 × 10^−2^ obtained from TGA measurements, which is consistent with the hypothesis of PB formation by direct electron transfer from SWCNTs to Fe^3+^.

**Fig. 7:**
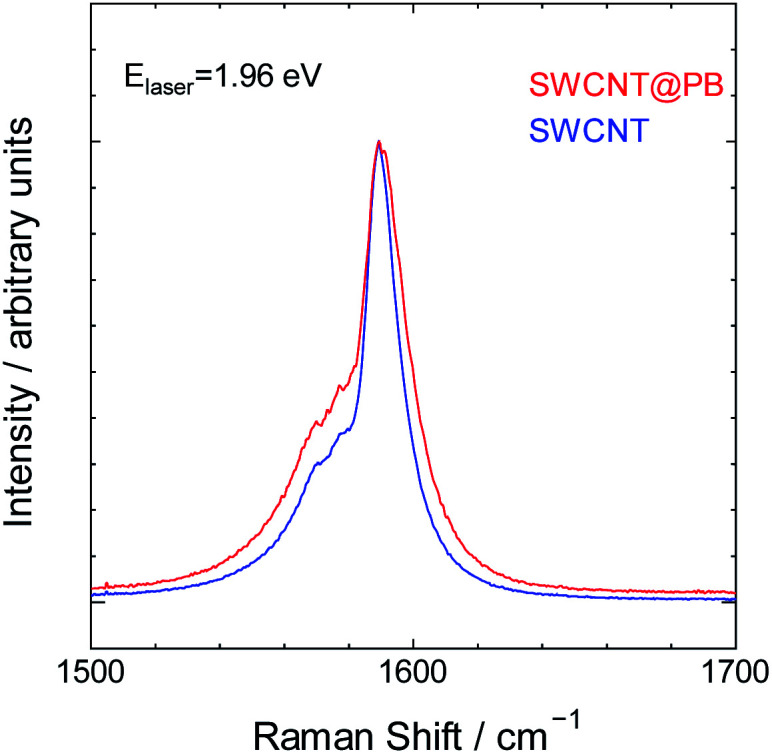
Raman spectra for bare SWCNTs and SWCNT@PB, excited with a He–Ne laser.

Finally, we characterized the bare SWCNTs and SWCNT@PB using Raman spectroscopy for insight into the charge carrier levels in both and the possibility of electron transfer. The Raman spectrum of SWCNTs comprises characteristic peaks that depend on the electronic structure of the SWCNTs.^57,58^ These peaks change in intensity, location, shape and other characteristics upon electron transfer to or from the tubes.^59–66^ The spectra for bare SWCNTs and SWCNT@PB are shown in Fig. 7 for the region where the double component G band is located. This peak appears due to bond stretching of all pairs of sp^2^ atoms in polyaromatic hydrocarbons.^67^ The two components of this band in SWCNTs are the G^+^ band around 1580–1590 cm^−1^ and the G^−^ band around 1560–1570 cm^−1^. The spectra in our study were collected using a He–Ne laser with an energy of 1.96 eV, which is close to the value of the energy transition between the second set of van Hove singularities in the DOS profile of the (21,5) SWCNTs (also known as *E*^M^_22_). This causes the excitation laser to prominently probe the characteristic peaks of the metallic SWCNTs in our sample, which is seen from the lower component of the G band that appears as an asymmetric peak assuming the so-called BWF line shape characteristic of metallic SWCNTs and graphite intercalation compounds.^57,68^ The intensity of the peak is higher and the BWF line shape is more asymmetric for SWCNT@PB compared to that of bare SWCNTs. This indicates higher levels of charge carriers (in this case positively charged holes) in the metallic tubes in SWCNT@PB compared to the bare SWCNTs, and further supports the hypothesis of direct electron transfer from SWCNTs and their role as the reducing agent in the PB synthesis reaction.

**Fig. 8:**
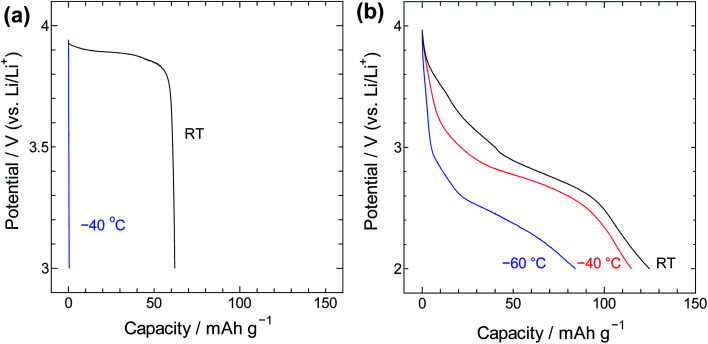
The discharge curves for (a) LiCoO_2_ and (b) SWCNT@PB.

For insight into the material's energy storage behaviour, the SWCNT@PB composite was used as a cathode material in a lithium-ion battery setting to evaluate its low-temperature performance. Constant current charge/discharge measurements were performed using SWCNT@PB and for comparison, LiCoO_2_ that is a common cathode material for lithium ion batteries. The results are shown in Fig. 8 and show that while the common cathode fails to work at −40 °C, the SWCNT@PB composite electrode continues to charge and discharge at temperature as low as −60 °C.

**Fig. 9:**
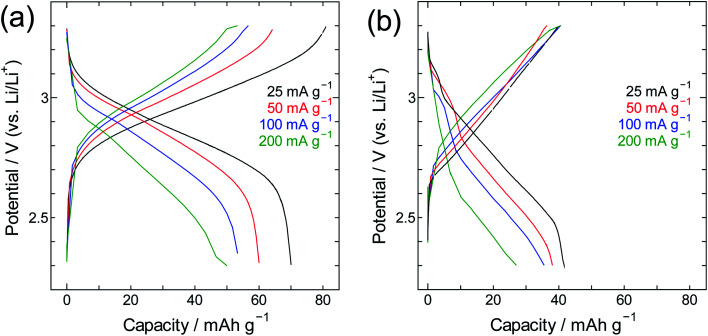
Charge/discharge curves for PB crystals at (a) room temperature and (b) −40 °C.

**Fig. 10:**
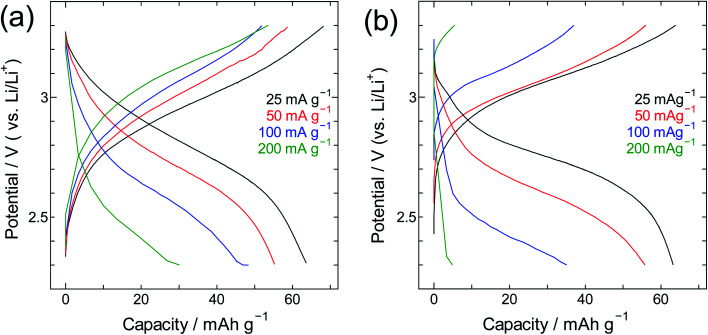
Charge/discharge curves for the SWCNT@PB composite at (a) room temperature and (b) −40 °C.

The performance of SWCNT@PB was also compared to that of crystalline PB (Fig. 9 and 10). Here one can see that for crystalline PB (Fig. 9) the slope of the charge/discharge curve increases visibly with current density at room temperature, *versus* minimally at −40 °C. This shows the effect of low diffusion kinetics at low temperature that results in consistently low capacity values in the whole current density range. The trend for the SWCNT@PB (Fig. 10) shows superior low-temperature performance compared to crystalline PB at low-to-moderate current density, but does not seem to cope as efficiently at higher current densities. This raises the question of the stability of the composite at high rates, and necessitates further work to optimize the synthesis conditions, product properties, and high rate performance. Nonetheless and even before optimization, the SWCNT@PB demonstrates advantage over crystalline PB and LiCoO_2_, performing well at temperature as low as −60 °C and maintaining a coulombic efficiency above 90% for 100 cycles (Fig. S2†).

## Conclusions

The synthesis of Prussian blue on SWCNTs can proceed by direct and spontaneous electron transfer from SWCNTs to the precursor material. *In situ* QCM tracking of the synthesis reaction showed that the formation of PB is instantaneous upon the addition of the precursor to the solution and that photoirradiation did not seem to contribute to additional reactions in our study. The number of electrons available from the SWCNTs used in our study was shown to be sufficient for the formation of PB with the content determined using TGA measurements, and Raman spectroscopy measurements showed higher hole density on the spectrum of the SWCNTs in the composite compared to that of bare SWCNTs. The findings of our study serve as a proof of concept for SWCNT Fermi level chemistry and open the door for future synthesis processes in which SWCNTs are designed to catalyse the desired reaction.

## Author contributions

The manuscript was written through contributions of all authors and they have all given approval to the final version of the manuscript.

## Conflicts of interest

There are no conflicts to declare.

## Supplementary Material

NA-004-D1NA00739D-s001
