# Supplementary material for: Single-walled carbon nanotubes as a reducing agent for the synthesis of a Prussian blue-based composite: a quartz crystal microbalance study
Source: Nanoscale Adv. 2021 Nov 24;4(2):510–20. doi: 10.1039/d1na00739d (PMC9417717; doi:10.1039/d1na00739d)
Supplement: NA-004-D1NA00739D-s001 [file NA-004-D1NA00739D-s001.pdf]

Electronic Supplementary Material (ESI) for Nanoscale Advances.
This journal is © The Royal Society of Chemistry 2021

Single-walled carbon nanotubes as reducing agent for the synthesis of Prussian blue-based composite: a quartz crystal microbalance study

Yosuke Ishii[1]*, Ayar Alzubaidi[1]*, Yoshimitsu Taniguchi[1], Shinya Jindo[1], Shinji Kawasaki[1]*

Electronic Supplementary Information

Table S1 The materials used in the present study

Material	Manufacturer
Ferric chloride ($FeCl_3$)	Wako Pure Chemical Corporation Ltd.
Potassium ferricyanide $K_3[Fe(CN)_6]$	Kishida Chemical Co., Ltd.
Single-walled carbon nanotube SWCNTs (MEIJO eDIPS)	Meijo Nano Carbon Co.,Ltd.
lithium bis (trifluoromethanesulfonyl) imide (LiTFSI) 1M	Kishida Chemical Co., Ltd.
2-methyltetrahydrofuran (2-methyl THF)	Sigma-Aldrich
Prussian blue (PB) crystal powder	Sigma-Aldrich
N-methyl-2-pyrrolidone (NMP)	Wako Pure Chemical Industries, Ltd.
lithium metal	Sigma-Aldrich
$LiCoO_2$	Sigma-Aldrich

SWCNT treatment

The impurities likely to be found on the surface of SWCNTs include the Fe catalyst particles remaining from the synthesis process, surrounded by depositions of amorphous carbon particles. To purify the sample, we first performed heat treatment in air at 350 °C for 30 minutes to remove amorphous carbon and expose the Fe particles. Then we washed the sample with10% HCl three times to remove the Fe particles.

Annealing treatment was performed to improve the crystallinity of the sample and remove any defects band chemical groups that preexisted on the surface or were induced by the purification treatments. The annealing was carried out by heat treatment of the sample at 1200 °C for 6 hours under vacuum (<1 Pa). Finally the end caps of the tubes in the sample were removed by heat treatment at 580 °C in air for 30 minutes. The decapping temperature was determined from thermogravimetric (TGA) analysis, performed using a SHIMADZU TGA-50 analyzer. The samples were heated to 1000 °C at a rate of 5 °C /min, under dry air flow of 100 mL /min.

To obtain the electronic density of states (DOS) profiles for SWCNTs in the sample, we used the Kataura plot that provides the DOS data for SWCNTs of different sizes and chiralities. The chirality of a SWCNT is given by the integer pair (n,m) called the chiral index. SWCNTs are metallic when (n-m)/3 is an integer, and semiconducting otherwise. We obtained two proxy DOS profiles for the (21,15) metallic and (21,16) semiconducting SWCNT as seen in **Figure S1**, in which the x-axis shows the density of states per one carbon atoms, and the y-axis shows valance and the conduction band energy levels with Fermi level taken as the point of 0 energy.

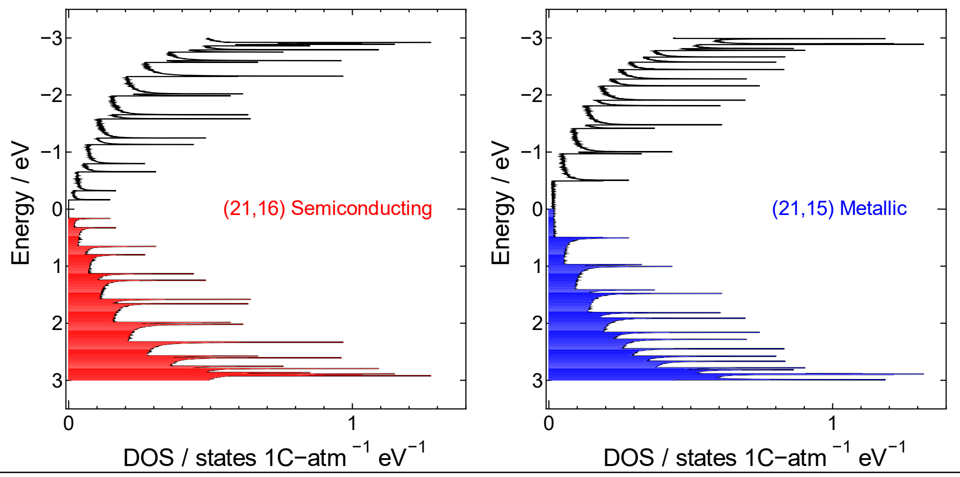

Figure S1. Electronic density of states for the candidate chiralities for sample in the present study

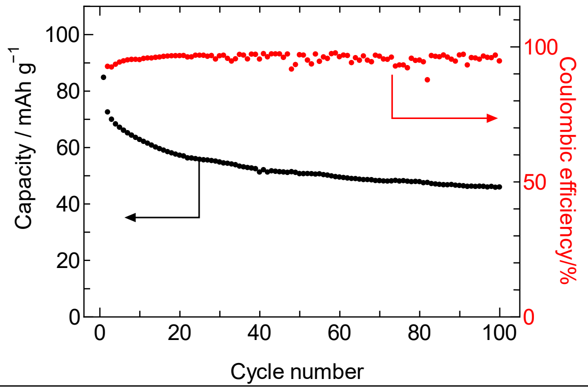

Figure S2. Cyclability and coulombic efficiency of the SWCNT@PB composite electrode
